# Deep origin and gradual evolution of transporting tissues: Perspectives from across the land plants

**DOI:** 10.1093/plphys/kiac304

**Published:** 2022-07-29

**Authors:** Sjoerd Woudenberg, Jim Renema, Alexandru M F Tomescu, Bert De Rybel, Dolf Weijers

**Affiliations:** Laboratory of Biochemistry, Wageningen University, Wageningen, The Netherlands; Ghent University, Department of Plant Biotechnology and Bioinformatics, Ghent 9052, Belgium; VIB Center for Plant Systems Biology, Ghent 9052, Belgium; Department of Biological Sciences, California State Polytechnic University–Humboldt, Arcata, California 95521, USA; Ghent University, Department of Plant Biotechnology and Bioinformatics, Ghent 9052, Belgium; VIB Center for Plant Systems Biology, Ghent 9052, Belgium; Laboratory of Biochemistry, Wageningen University, Wageningen, The Netherlands

## Abstract

The evolution of transporting tissues was an important innovation in terrestrial plants that allowed them to adapt to almost all nonaquatic environments. These tissues consist of water-conducting cells and food-conducting cells and bridge plant–soil and plant–air interfaces over long distances. The largest group of land plants, representing about 95% of all known plant species, is associated with morphologically complex transporting tissue in plants with a range of additional traits. Therefore, this entire clade was named tracheophytes, or vascular plants. However, some nonvascular plants possess conductive tissues that closely resemble vascular tissue in their organization, structure, and function. Recent molecular studies also point to a highly conserved toolbox of molecular regulators for transporting tissues. Here, we reflect on the distinguishing features of conductive and vascular tissues and their evolutionary history. Rather than sudden emergence of complex, vascular tissues, plant transporting tissues likely evolved gradually, building on pre-existing developmental mechanisms and genetic components. Improved knowledge of the intimate structure and developmental regulation of transporting tissues across the entire taxonomic breadth of extant plant lineages, combined with more comprehensive documentation of the fossil record of transporting tissues, is required for a full understanding of the evolutionary trajectory of transporting tissues.

## Teaser

Combining fossil records and recent molecular research provides insights into the origin and evolutionary progress of transporting tissue development in land plants.

## Introduction

Land plants evolved more than 500 million years ago from a green algal ancestor (Morris et al., 2018; Strother and Foster, 2021). During evolution, terrestrial plants became less dependent on water compared to their aquatic ancestors. One of the major evolutionary innovations involved in this process was the development of water- and food-transporting tissue systems throughout the plant (de Vries and Archibald, 2018). These transport systems link the roots in the soil to the leaves in the atmosphere, solving one of the biggest tradeoffs in plant life history by allowing for high rates of gas exchange while remaining hydrated. Together, these facilitated the evolution of a wide diversity of terrestrial plants including sky-scraping trees and serpentine vines.

All terrestrial plants have plasmodesmata that connect the cytoplasm of adjacent cells, but a dedicated tissue system for long-distance transport of substance is estimated to be a million times more efficient for multicellular plants (Raven, 1977). Most plant taxa have some type of dedicated transporting system, referred to here as water-conducting cells (WCCs) and food-conducting cells (FCCs). Additionally, one of the main factors in the evolution of WCC was the appearance of lignin, granting mechanical strength, hydrophobicity, and flexibility (Boyce et al., 2004). Lignin is even argued to be crucial for xylem cell adaptability and physiology at a cellular level (Ménard et al., 2021).

The importance of these WCCs that contain lignin is reflected by the naming of an entire clade after them, which contains 95% of all extant plant species: the tracheophytes or vascular plants. Tracheophytes contain specialized WCC, called tracheids, and FCC, named sieve elements. These two cell types are the functional parts of the tracheophyte transporting tissues xylem and phloem, respectively. Together with additional supporting tissues, they make up the vasculature or the vascular tissues (Scarpella and Helariutta, 2010).

**Figure 1:**
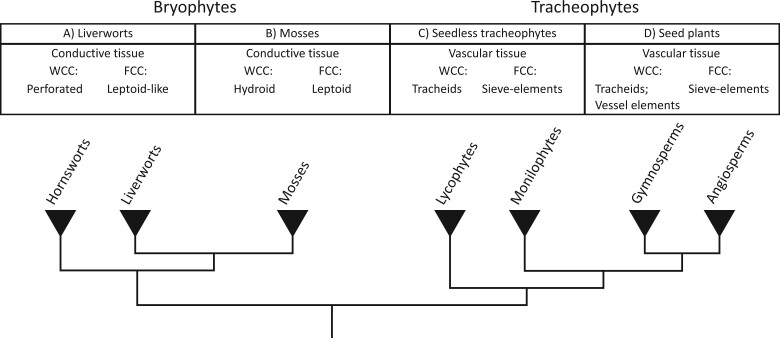
Terminology and phylogenetic relationships. The land plant lineages contain different transporting tissues and cell types for the transport of water, nutrients and assimilates. Due to taxonomic and historical reasons, they have a distinct nomenclature. A, In liverworts all tissues involved in transport are called conductive tissues, collectively, and they can be composed of hydroid-like WCCs and leptoid-like FCCs, if present. B, In mosses, conductive tissues are also composed of WCC (hydroids) and FCC (leptoids), but these are more specialized and more broadly occurring compared to liverworts. Some species that contain WCC lack the diagonal end wall of hydroids (Figures 2 and 3). C, In seed-free tracheophytes (lycophytes, ferns) all tissues involved in transport are called vascular tissues and include xylem and phloem cell types. The generalized WCC in the xylem are tracheids with secondary cell wall thickenings, while the generalized FCC in the phloem are sieve cells. D, Angiosperms (and some gymnosperms) evolved vessel elements (a more specialized type of WCC with completely perforated end walls and increased girth) in the xylem, and sieve elements (a more specialized type of FCC with sieve plates in the end walls) in the phloem.

The bryophyte lineages form the remaining 5% of extant species that lack lignified WCC. Yet, bryophytes do possess conductive tissues (Tansley and Chick, 1901; Hébant, 1977), which consist of perforated or imperforated WCC, called hydroids, and FCC, named leptoids (Ligrone et al., 2000; Figure 1). The difference in nomenclature compared to tracheophytes is based on historic and morphological reasons but can make comparative discussions somewhat confusing. We have included a glossary (Table 1) to clarify some of the terminology used. Additionally, the sparse sampling of plant species throughout the evolutionary tree (especially bryophytes) and their fossil records (Bippus et al., 2022) results in considerable gaps in knowledge, contributing to a poor starting point for comparative anatomy.

**Table 1:** Glossary of terms used.

Term	Definition
Angiosperms	Flowering plants—seed plants in which the seeds are encapsulated within carpels, which are part of flowers
Bryophytes	Nonvascular free-sporing plants that include hornworts, liverworts, and mosses. The haploid gametophytic phase dominates their life-cycle. The three bryophyte lineages are recovered as a clade in some phylogenetic analyses but not in others
Embryophytes	Land plants—the clade including bryophytes and vascular plants, derived from charophycean ancestors and characterized by a diplohaplontic life-cycle that includes multicellular haploid and diploid phases (gametophyte and sporophyte, respectively)
Ferns	Group of seed-free vascular plants of debated membership. Phylogenies including exclusively extant plants equate them with the monilophyte clade, which is sister to the seed plant clade and consists of horsetails, psilotophytes (*Psilotum* and *Tmesipteris*) and all other nonlycophyte seed-free tracheophytes. In phylogenies that include fossils, the extant monilophytes are polyphyletic
Gymnosperms	Seed plants in which the seeds are not fully enclosed in any structures, or all the nonflowering seed plants. Living gymnosperms include the conifers, *Ginkgo*, cycads, and gnetophytes
Hornworts	Small clade of bryophytes with thalloid gametophytes and a distinctive sporophyte that is entirely sporangial in nature and has prolonged growth from a basal meristem. Include the genus *Anthoceros*
Liverworts	Clade of bryophytes with thalloid or leafy gametophytes bearing single-celled rhizoids and with ecolumellate sporangia. *Marchantia polymorpha* is the best studied liverwort
Lycophytes	Clade of seed-free vascular plants and sister group to the clade including all seed plants and ferns; includes spike mosses (*Selaginella*), clubmosses, and *Isoetes*
Mosses	Largest clade of living bryophytes including taxa like *Sphagnum* and *Physcomitrium patens*. Mosses have leafy gametophytes and sporophytes with elongated stem-like seta and columellate sporangia
Seed plants	Gymnosperms and angiosperms—clade of heterosporous plants in which the indehiscent megasporangium is covered in an integument. Also known as spermatophytes
Seed-free tracheophytes	A paraphyletic group including all free-sporing tracheophytes (lycophytes, ferns, horsetails, psilotophytes)
Tracheophytes	Vascular plants—clade of plants possessing water-conducting tissue (xylem) that contains tracheids and food-conducting tissue (phloem) that contains sieve cells. They include seed plants and seed-free tracheophytes and have a diplohaplontic life-cycle dominated by the diploid sporophytic phase

The classical view of transporting tissue evolution assumed an innovation in tracheophytes that has no counterpart in bryophytes. However, the occurrence of conductive tissues in some bryophytes, along with fossil discoveries (Edwards et al., 2022) and recent re-evaluations of phylogenetic relationships among the bryophyte lineages and vascular plants (e.g. Puttick et al., 2018; Bell et al., 2020; Donoghue et al., 2021) have sparked discussions about the traditional view on transporting tissue evolution among embryophytes (Edwards et al., 2022; Tomescu, 2022). These discussions that consider alternative hypotheses of relationships among the main embryophyte lineages have important consequences for the rate and nature of evolutionary events that gave rise to conductive and vascular tissues, as well as for the underlying genetic changes associated with these events. For example, while predominant views have focused on the gain of vascular tissues as a novelty in the tracheophyte lineage, one might have to consider an earlier origin in the common ancestor of embryophytes and subsequent losses in some bryophyte lineages.

In this opinion paper, we set out to explore what extant bryophytes and the fossil record can teach us about the unique features of tracheophyte vasculature and how this relates to recent molecular insights. We attempt an integrated overview that compares the transporting tissues of bryophytes and tracheophytes and integrates data from the early embryophyte and tracheophyte fossil record, to better understand the evolutionary history of transporting tissues.

## The diversity of transporting tissues among extant plants

**Figure 2:**
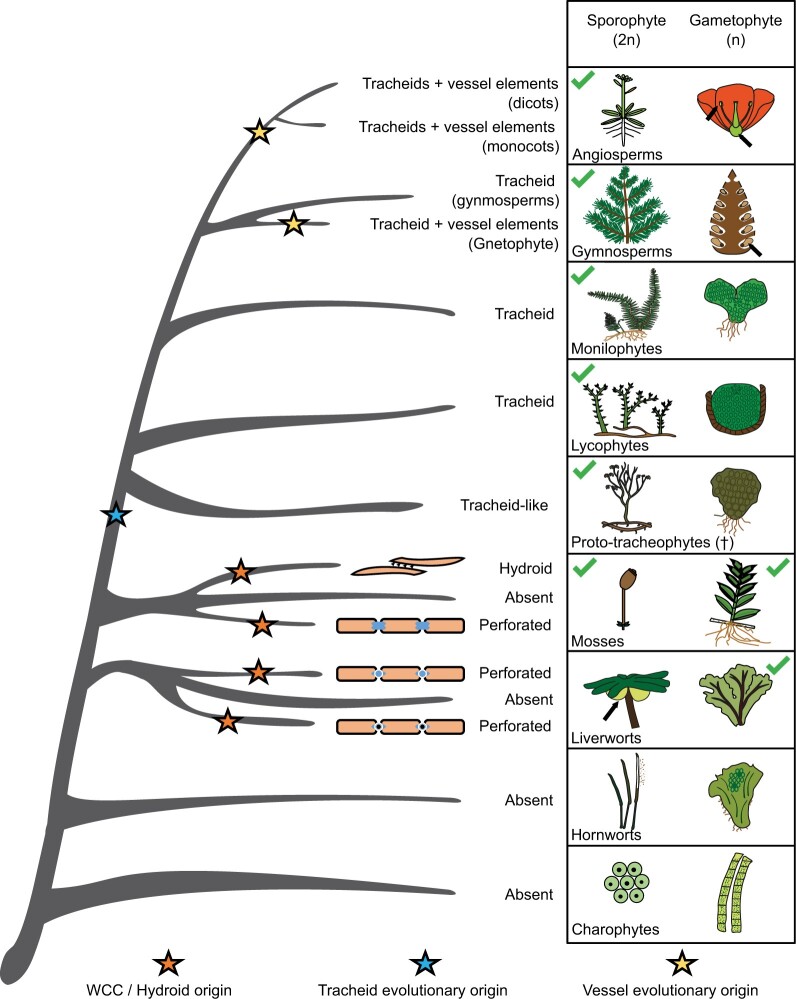
Evolutionary overview of WCCs throughout the land plant lineage and their occurrence between the haploid (gametophyte) and diploid (sporophyte) phases depicted by green checkmarks. Arrows point at the sporophyte or gametophyte if highly reduced. Representative species are chosen for each clade. Bryophyte listing based on Hébant (1977) and Ligrone et al. (2000, 2012).

Conductive tissue is widely spread among different bryophytes, most of which are seen in liverwort and moss species (Figure 2). Mosses can possess hydroids and/or leptoids, while liverworts can possess perforate WCC and leptoid-like cells. Conductive tissues are not restricted to only gametophytes or only sporophytes, since some bryophytes show WCC and FCC in both generations (Renzaglia et al., 1997; Ligrone et al., 2012). In contrast to the variable occurrence of conductive tissues in bryophytes, all tracheophytes contain a vasculature, consisting of xylem with tracheids and phloem with sieve elements as transporting tissues.

Tracheids are defined as elongated, nonliving cells, which have a secondary cell wall with differential thickenings. They are connected to other cells by openings in the secondary cell wall, called pits (Decombeix et al., 2019). Bryophyte hydroids are similarly elongated and dead upon functioning and can also contain variable cell wall thickenings; albeit lacking the tracheophyte-specific lignin polymer. Most bryophyte species, however, possess thin-walled hydroids (Ligrone et al., 2000), which do not play a role in mechanical support, in contrast to tracheids. Therefore, bryophytes often contain specialized reinforced and elongated cells, known as stereids that perform a structural role. The major differences between WCC in tracheophytes and bryophytes seem to be related to cell-to-cell connection (Figure 2) and composition and thickening of the cell wall (see Biochemical properties). Cell-to-cell connections in all tracheids involve diagonal end walls with pits, while numerous pits also line their side walls enabling transfer between neighboring tracheids. Bryophyte WCCs are not so uniform in their appearance as some have diagonal end walls (mosses), while others have horizontal end walls (liverworts; Ligrone et al., 2000, 2012).

Sieve elements and leptoids share a common classifier as elongated, thick-walled cells with an increased number of intercellular connections (van Bel et al., 2002). Similar to sieve elements, leptoids have some form of sieve areas with enlarged plasmodesmatal openings (Scheirer, 1990). Bryophyte leptoids differ in their presence of a small vacuole, lacking plastids, and having a microtubular cytoskeleton and intact mitochondria. Leptoids thus seem to depend less on their surrounding cells for functionality, which is a characteristic of the angiosperm sieve tube element and companion cell complex. Extant seed-free tracheophytes and gymnosperms show sieve elements which shared characteristics of both leptoids and angiosperm sieve elements (Evert, 1990; van Bel et al., 2002), suggesting a gradual evolution and deep origin of these cell types.

**Figure 3:**
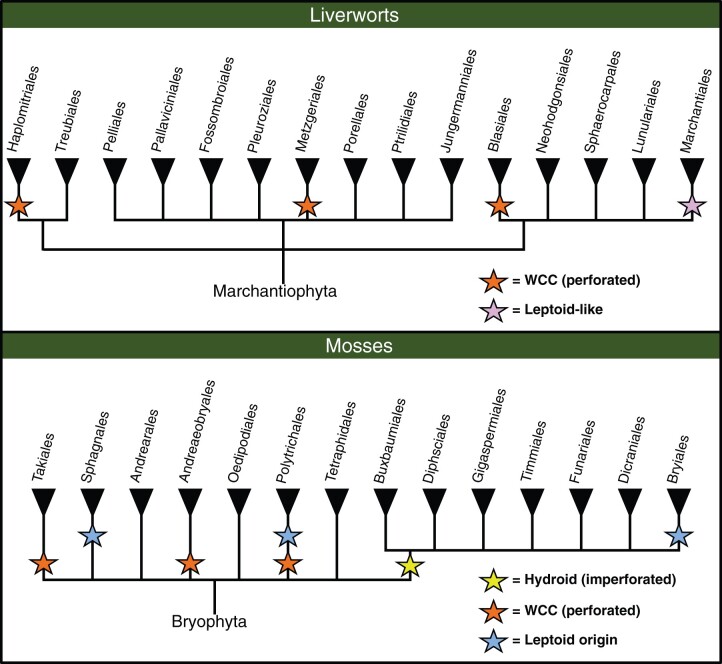
Overview of WCCs and FCCs presence in bryophyte clades. Upper panel depicts liverwort clades and the bottom panel depicts mosses. Based on Ligrone et al. (2000, 2012).

The perceived intergradations between the characteristics of transporting tissues across land plants begs the question of what are ancestral and which are derived traits in these tissues. The absence of conductive tissue in most bryophyte families (Figure 3) has long been considered the ancestral trait of land plants (Ligrone et al., 2000). In this scenario, different lineages would have converged upon similar cell types and tissues. In this context, the presence of sieve element-like FCC in phylogenetically unrelated brown algae (Sykes, 1908) suggests the broad potential for the evolution of transporting tissues in autotrophic organisms. Alternatively, the presence of transporting tissue could be the ancestral trait for the bryophyte–tracheophyte clade, with numerous losses in extant species due, for example, to selective pressures brought about by competition with tracheophytes or by adaptation to changing environments. Future discoveries and studies of early bryophyte fossils (Tomescu et al., 2018) and of fossils that bridge the bryophyte–tracheophyte gap (e.g. Edwards et al., 2022), as well as improved understanding of the genetic and molecular programs instructing conductive and vascular tissue development, along with the deep phylogenetic relationships of the different lineages, will likely help to further resolve the origin and sequence of character evolution in transporting tissues.

## The fossil record suggests stepwise evolution of transporting tissues

**Figure 4:**
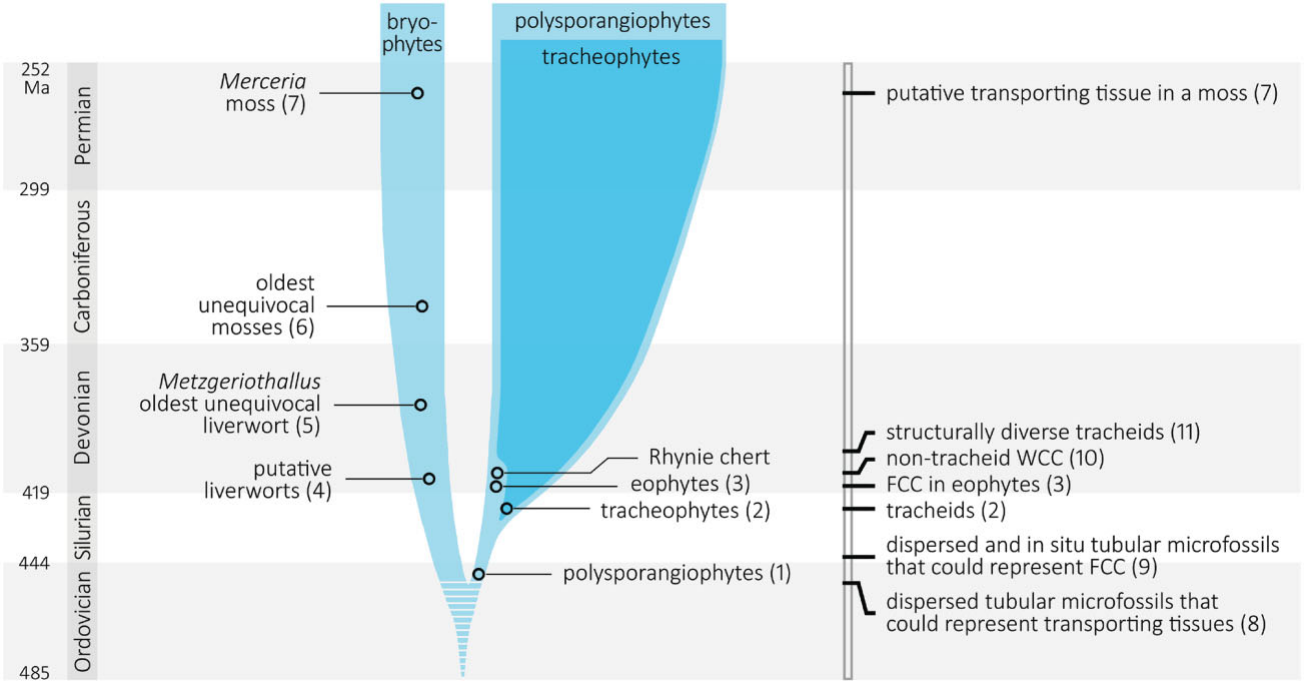
Summary of the early fossil record of transporting tissues. The timeline of oldest fossil occurrences representing different plant lineages (left) is shown in parallel with a timeline of the oldest evidence for different transporting cell or tissue types (right). Numbers correspond to references as follows: 1, Salamon et al. (2018); 2, Edwards and Davies (1976); 3, Edwards et al. (2022); 4, Tomescu et al. (2018); 5, Hernick et al. (2008); 6, Hübers and Kerp (2012); 7, Smoot and Taylor (1986); 8, Edwards and Wellman (2001); 9, Pratt et al. (1978); Burgess and Edwards (1991); Niklas and Smocovitis (1983); 10, Edwards (1993, 2003); 11, Kenrick and Crane (1991); Edwards (1993); Cascales‐Miñana et al. (2019).

Axial fossils from the earliest Early Devonian (415 mega annum (Ma)) possess transporting tissue consisting of cells that are most similar to those of modern bryophyte FCC (Edwards et al., 1995, 2022). Those fossils, recently assigned to an early land plant group referred to as eophytes (Edwards et al., 2022) lack any evidence of WCCs (Figure 4). Dispersed tubular microfossils that can be traced to the early Silurian (440 Ma; Pratt et al., 1978; Burgess and Edwards, 1991) are thought to derive from the same type of plants (Edwards et al., 1995), wherein they would have performed the same function. Supporting this, a small early Silurian axial fragment possesses similar cells that probably formed transporting tissue (Niklas and Smocovitis, 1983), most likely FCC (Edwards et al., 1995). Together, these fossils indicate that the earliest type of specialized transporting tissue consisted exclusively of FCC and was present in land plants at least as early as 440 Ma ago.

While these fossils provide a minimum age for the origin of FCC-based transporting tissue, the phylogenetic depth of this origin cannot be ascertained using available data because of a number of issues. Based on their branched morphology, the eophytes are regarded as stem-group polysporangiophytes (Edwards et al., 2022). The polysporangiate condition (e.g. the branched sporophyte) is thought to have preceded the evolution of vascular tissues in a clade that excludes the bryophytes (Mishler and Churchill, 1985; Edwards et al., 2022) and the oldest polysporangiophyte fossils go farther back in time than the eophytes, to 445 Ma, latest Ordovician (Salamon et al., 2018). However, because of their preservation, we do not know if these oldest polysporangiophytes possessed any type of transporting tissue. Nevertheless, the existence of polysporangiophytes as old as 445 Ma implies that the sister group of the polysporangiophyte clade was also present on land. Whether this sister group was the common ancestor of a clade comprising all three bryophyte lineages, only two of them, or simply one of the three bryophyte lineages, is still debated (because of the lack of unequivocal phylogenetic resolution among the main embryophyte lineages (Puttick et al., 2018; Bell et al., 2020). Either way, this means that bryophyte-type plants were present as early as 445 Ma in the Late Ordovician. Thus, the possibility that some of the FCC-like dispersed microfossils reported from early Silurian and younger rocks are derived from bryophyte-type plants cannot be excluded. In fact, using evidence from comparative anatomy and development, Edwards et al. (2022) have proposed a common origin of bryophyte and tracheophyte FCC. This would imply that the ancestral embryophyte possessed FCC, in which case the shared characteristics of bryophyte and tracheophyte FCC—elongated shape, enlarged plasmodesmata, breakdown of the vacuole, and symplastic transport (Ligrone et al., 2000)—provide a minimum set of features that could have characterized the ancestral FCC.

Among FCC-equipped polysporangiophytes, the most derived type of WCC—tracheids—had evolved by the late Silurian 425 Ma ago (Figure 4; Edwards and Davies, 1976). The preferential preservation of xylem tracheids in all plant fossils thereafter demonstrates that these WCC had evolved a cell wall layer impregnated with specialized chemical compounds that made them resistant to degradation, although it is difficult to prove that these compounds had the exact chemistry of modern lignin. As a consequence of this, the absence of tracheids with degradation-resistant walls older than the late Silurian indicates that this type of WCC did not evolve much earlier than 425 Ma ago. By about 410–400 Ma ago in the Early Devonian, the wall thickenings recorded in the tracheids of different vascular plant groups (Kenrick and Crane, 1991; Edwards, 1993; Cascales‐Miñana et al., 2019) had attained a level of structural diversity unequalled since. If the different structural types documented in the Early Devonian represent successive stages in the evolution of development of tracheid secondary wall thickenings, as hypothesized by Cook and Friedman (1998), then the subsequent reduction in the diversity of secondary wall thickenings to only two—represented by modern lycophyte and euphyllophyte tracheids—would be the result of post-Early Devonian extinctions.

Nontracheid WCCs are reported in approximately 409-Ma-old polysporangiophytes of the Early Devonian Rhynie Chert: the protracheophyte *Aglaophyton* with elongated cells possessing thin walls with reticulate thickenings, which were probably not lignified (Boyce et al., 2003); and *Nothia* with WCC possessing uniformly thickened walls with possibly bilayered organization (Edwards, 1993, 2003). Although these nontracheid WCCs have been compared with those of bryophytes, they have no exact counterparts among bryophytes (Edwards, 1993). Beyond these Early Devonian occurrences, the fossil record of nontracheid WCC cannot be reconstructed from the available data. For instance, some of the dispersed tubular microfossils that go back to the late Ordovician (445 Ma ago; Edwards and Wellman, 2001) may be embryophyte WCC (or FCC) but cannot be ascertained as such until found preserved within plant tissues, because nontracheid WCC—whether bryophytic or polysporangiophytic—lack unequivocal diagnostic features.

In summary, our current understanding of the fossil record and phylogeny of early embryophytes and polysporangiophytes suggests a deep origin and stepwise evolution of transporting tissues. The earliest such tissues seem to have been food transporting and may have evolved as deeply as a common ancestor of bryophytes and tracheophytes. The eophytes, which possess FCC but lack WCC, suggest that the latter evolved later among polysporangiophytes; likewise, the WCC of protracheophytes suggest xylem tracheids evolved by sequential acquisition of characters in WCC (Cascales‐Miñana et al., 2019). Chronologic discrepancies between this hypothetical sequence of transporting tissue evolution and its phylogenetic progression among polysporangiophytes (Tomescu, 2022) are probably a reflection of huge gaps in the exploration of the fossil record and scale-related difficulties in evaluating fossil evidence. To date, only a precious few fossil assemblages have delivered plant fossils with tissue-level preservation of ages relevant to questions related to the deep evolution of transporting tissues. Concurrently, detailed comparisons that can lead to empirically supported inferences on the function of fossil cells and tissues require observation of structural details at very fine anatomical scales. In turn, this requires exceptional cellular preservation of plant fossils, which further narrows down the number of known fossil assemblages that can be queried to address the evolution of transporting tissues.

Perhaps the most crucial unknowns—and accordingly the biggest potential source of breakthroughs—are associated with the bryophyte fossil record, which is extremely sparse in its deepest reaches. The oldest unequivocal bryophyte fossils (exclusively preserved spores) are only ∼385 Ma old (Early Devonian), with older records bearing increasingly more questionable bryophyte affinity (Tomescu et al., 2018). Important for our discussion, bryophyte fossils that preserve internal tissues are only 255 Ma old (Smoot and Taylor, 1986), much too young to have any bearing on questions of deep transporting tissue evolution. Thus, if bryophytes are at least as old as the polysporangiophytes (445 Ma), all major questions related to the earliest players and steps of the evolution of their transporting tissues and the bryophyte–tracheophyte transition remain unanswered, for now, pending denser sampling of the deep bryophyte fossil record; along with them, our understanding of what the early bryophyte FCC and WCC looked like. Likewise, it is currently not possible to confidently state whether water-transporting tissues have a single common origin or evolved independently in the polysporangiophytes and various bryophyte lineages.

## Organization and patterning of transporting tissues

Despite the broad histological diversity of each of the water-transporting and food-transporting tissues across embryophytes, these transport systems always occupy the central regions of the plant organs (Spencer et al., 2021; Tomescu, 2021). Moreover, in the arrangement of transporting tissues relative to each other, the WCCs are always positioned closer to the center of the axis (whether stem or root) than the food-transporting cells, if present (Scarpella and Helariutta, 2010). This consistency in the relative positioning of the two tissue types across plant organs and lineages suggests strongly conserved developmental or physical constraints responsible for the overall organization of transporting tissues along the radial axis of polarity. In line with this, the water transport cells are often thick-walled elements, or accompanied by stereids in some bryophytes, for mechanical support (Ligrone et al., 2000), hinting toward a conservation in plant body plan and the overall organization of transporting tissues.

In contrast, the actual patterning of transporting tissues observed among species and even within the different organs of a single plant is incredibly diverse. For example, in many tracheophytes, vascular patterning is strikingly different between shoots and roots. Tracheophyte roots all possess a protostele type of organization with a single central strand of xylem. Lycophytes also possess protostelic organization in their shoots (Gifford and Foster, 1989; Decombeix et al., 2019), while most fern shoots possess siphonosteles that feature a central pith and angiosperms shoots have a derived eustele consisting of discrete vascular strands each containing both xylem and phloem (Tomescu, 2021). Recently, a detailed analysis of the fern clade suggested that innovations in vascular organizations can mainly be traced back to body size, suggesting no direct selection on organization (Suissa and Friedman, 2022).

Nonetheless, the protostelic organization of roots is similar to that of bryophytes, which possess a central core of hydroids surrounded by leptoids. This shared organization suggests that both share ancestral mechanisms of tissue patterning. The connection between the two is provided by the earliest fossil tracheophytes, whose sporophytes were not differentiated into stems, leaves, and roots, and instead consisted of simple branched photosynthetic axes. On one hand, these axes, which had protostelic organization (e.g. Gensel and Andrews, 1984), were simple elaborations of the unbranched sporophytic axes of bryophytes (Rothwell et al., 2014). On the other hand, they were the basic organ type from which roots (and shoots) evolved in different lineages (Boyce, 2010), likely inheriting the same mechanisms of tissue patterning.

## Evolution of the molecular toolbox for transporting tissue development

In recent years, while molecular research on nonangiosperm models has gained traction, understanding of the molecular mechanisms underlying the development and differentiation of WCC and FCC has remained limited. This can largely be attributed to the choice of nonangiosperm models currently used: the popular liverwort model *Marchantia polymorpha* (further referred to as Marchantia) lacks any form of differentiated conductive tissues, while the commonly studied moss *Physcomitrium patens* lacks leptoids. Clearly, expanding this set of genetic/molecular model systems by addition of bryophytes that have a broader suite of conducting cells, as well as studies on lycophytes and ferns (such as *Selaginella moellendorffii* and *Ceratopteris richardii*) holds great promise for filling this gap in our knowledge. Genome and RNA-seq data (e.g. 1KP project) are becoming available for many more species. Together with the increasing use of single-cell technologies, including bryophytes (Kubo et al., 2019), this should help better define commonalities and differences in tissue types and their tissue-specific regulators and identity markers across species. Taken together, recent technological advances provide a unique starting point to understand the key innovations that allowed complex transporting tissue development.

**Figure 5:**
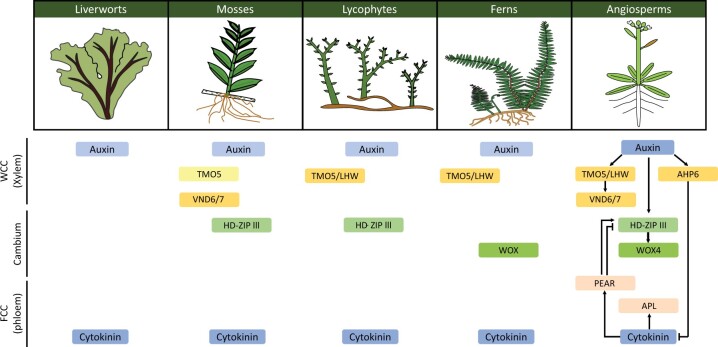
Overview of key vasculature regulators for specification and differentiation known from Arabidopsis [reviewed in De Rybel et al. (2016) and Ohtani et al. (2017)], and the orthologs of these genes in other taxa with assumed similar roles. ARABIDOPSIS HISTIDINE PHOSPHOTRANSFER PROTEIN 6 (AHP6), WUSCHEL RELATED HOMEOBOX 4 (WOX4), PHLOEM EARLY DOF (PEAR), and ALTERED PHLOEM DEVELOPMENT (APL) are not further discussed in the text and lack evolutionary characterization. WOX functioning in ferns based on Youngstrom et al. (2022). Note that Marchantia has no hydroids or leptoids and that little information is available for these bryophytes. Certainty about the functioning decreases from Arabidopsis to bryophytes (lighter coloring of the boxes).

In general, key processes and their associated protein families in vasculature development seem highly conserved throughout evolution (Figure 5; Floyd et al., 2006; Lucas et al., 2013; Xu et al., 2014; Bennett, 2015; Ohtani et al., 2017; Mutte et al., 2018; Wu et al., 2019; Lu et al., 2020; Mohanta et al., 2020). This is in line with the hypothesis that land plant innovation is mainly based on gene co-option rather than evolution of novelties (Bowles et al., 2020). However, gene families tend to increase in size within the tracheophyte lineage, compared to bryophytes. The NAM-ATAF1,2-CUC2 (NAC) and DNA binding with one finger transcription factor families show the most considerable increases, approximately 10-fold or higher, between bryophytes and angiosperms, corresponding with vascular tissue evolution (Bowman et al., 2017). Most genes involved in vascular development known from angiosperms have orthologs across embryophytes, suggesting the existence of a regulatory module that dates back to the earliest land plants (Bowles et al., 2022). However, in terms of functional characterization of such conserved genes there are big gaps in our knowledge, which preclude discussions of major aspects in the evolution of transporting tissue development.

In xylem, the three key processes of conducting cell differentiation are cell elongation (including cell wall loosening), cell wall thickening, and programmed cell death which are universal processes found throughout the land plant lineage. Moss hydroids share these characteristics (Hébant, 1977). The main requirement for producing a functional water-transporting system would be to coordinate these processes. Such coordination in *Arabidopsis thaliana* (hereafter referred to as Arabidopsis) prominently involves auxin and cytokinin signaling, whose interplay positions the xylem and phloem poles in the root (Bishopp et al., 2011; De Rybel et al., 2013, 2014, 2016). Extensive research into these hormonal pathways showed a strong conservation in all land plants. The nuclear auxin signaling pathway is deeply conserved in land plants (Mutte et al., 2018), and there are even uncharacterized homologs to key components of this pathway among the closest algal relatives of land plants (charophytes). The scenario for cytokinin signaling is very similar [reviewed in Rashotte (2021)]. However, the roles of these two signaling pathways in transporting tissue patterning have not been explored in depth outside of flowering plants. Similar to Arabidopsis vascular patterning, the *P. patens* cytokinin response was shown to be auxin-sensitive (Lavy et al., 2016; Rashotte, 2021), suggesting that this link may be ancient. *P. patens* PIN proteins [reviewed in Naramoto et al. (2022)] are expressed in the midvein of the gametophytic leaves, which contains hydroids (Viaene et al., 2014). However, auxin response reporters (e.g. *GH3*) do not show apparent signal in midveins (Bennett et al., 2014). Thus, while it is certainly possible that the hormonal control underlying patterning is conserved, there is no direct homology at this level, and roles of auxin and cytokinin in conductive tissue development in bryophytes largely remain an open question.

While analogies at the level of hormone signaling are unclear and not studied in detail, the role of the immediate signaling output has been explored in more depth. In Arabidopsis, the auxin-activated TARGET OF MONOPTEROS 5 (TMO5) transcription factor heterodimerizes with LONESOME HIGHWAY (LHW) to promote vascular cell proliferation through activating cytokinin biosynthesis (Ohashi-Ito and Bergmann, 2007; Schlereth et al., 2010; De Rybel et al., 2013, 2014; Ohashi-Ito et al., 2014). Homologs for both TMO5 and LHW are found in all three major bryophyte lineages. Bryophyte TMO5 orthologs were able to rescue Arabidopsis *tmo5* double mutant phenotypes, while LHW orthologs were unable to rescue *lhw* mutants. In *M. polymorpha* it thus appears these orthologs play independent roles (Lu et al., 2020). On this basis, it is proposed that the heteromeric interaction arose uniquely in the ancestors of tracheophytes, in which these two transcriptional regulators were redeployed for vasculature proliferation.

The examples of TMO5 conservation and, concurrently, proposed LHW neo-functionalization show the difficulty to identify conserved functions in transporting tissue development and genetic regulation, across wide evolutionary distances. Most studies focus on comparisons of gene expression domains between bryophytes and angiosperms while the two groups are highly divergent in terms of anatomy and life history. This is reflected in the low level of conservation of gene expression patterns between bryophytes and angiosperms (Szövényi et al., 2011). Nonetheless, HD-ZIP class III transcription factors, strongly associated with vascular tissue development in Arabidopsis (Emery et al., 2003), are likewise expressed in the *P. patens* leaf midrib (Yip et al., 2016), which possesses hydroids, as well as in *S. moellendorffii* vasculature (Floyd et al., 2006; Frank et al., 2015). In *P. patens*, suppression of HD-ZIP III expression causes defects in midrib formation. However, these are accompanied by pleiotropic defects in gametophore development (Yip et al., 2016), which makes inference of a role in conductive tissue development difficult. HD-ZIP III genes are regulated by the deeply conserved miRNA165/166 (Tsuzuki et al., 2016; Yip et al., 2016; Yadav et al., 2021), and this suggests that a miRNA/HD-ZIP III module may broadly operate in controlling transporting tissue development.

Vascular development in Arabidopsis prominently features intercellular movement of hormones, proteins, and small RNA’s [reviewed in De Rybel et al. (2016)]. In addition to auxin and cytokinin, these include SHORT-ROOT (Nakajima et al., 2001; Cui et al., 2007) and AT-hook motif nuclear-localized (Zhou et al., 2013) proteins, as well as the previously mentioned miRNA165/166 (Carlsbecker et al., 2010). The extensive incorporation of mobile signals in the development of transporting tissues seems less pronounced in bryophytes and seed-free tracheophytes. Plasmodesmata seem to be differently wired in bryophytes and ferns (Ligrone and Duckett, 1998; Imaichi and Hiratsuka, 2007; Imaichi et al., 2018), while tRNA and mRNA mobile signals seem absent from bryophytes (Fabres et al., 2021). Possibly, mobile signals and vascular tissue development co-evolved; facilitating the increasing complexity of tracheophyte vascular tissues compared to the less elaborate bryophyte conductive tissues.

## Cell identity specification and differentiation in transporting tissues

The regulation of cell position, identity, and tissue patterning is likely to be strongly adapted to unique anatomies and specific functional needs. In contrast, the functional differentiation leading to conducting cells may be more similar between different lineages, given the constraints that function imposes on cellular properties. As the key differentiation regulators are being identified by molecular and genetic studies in Arabidopsis and other tracheophytes, comparative analyses indicate that certain aspects of the regulation of conducting cell differentiation are conserved among lineages. NAC-domain containing VASCULAR-RELATED NAC-DOMAIN (VND) proteins are involved in xylem differentiation and are sufficient to ectopically trigger xylem-like secondary cell wall formation in tracheophytes (Kubo et al., 2005). A study on *P. patens* showed that its VND orthologs, named VNS, are expressed in gametophore midribs and are necessary for hydroid and stereid differentiation and functioning. Triple *vns* mutants show disrupted hydroid and stereid development, which reduces water-conducting capacity but without alteration of the overall growth habit under standard growth conditions. Additionally, expression of Arabidopsis VND and *P. patens* VNS factors showed known downstream target genes to be conserved (Xu et al., 2014). This evidence strongly suggests a common origin, rather than homoplasy between hydroids and tracheids.

Besides these striking similarities, one substantial difference is that *P. patens* hydroids do not contain a pronounced secondary cell wall (Xu et al., 2014). Moreover, secondary walls structurally equivalent to those of tracheophyte tracheary elements have not been demonstrated in the hydroids of any other bryophyte species (Ligrone et al., 2000). However, the NAC orthologs of *P. patens* and *Sphagnum palustre* can induce secondary cell wall thickening in Arabidopsis and *Nicotinana benthamiana*, respectively (Xu et al., 2014; Terada et al., 2021). Although limited to a single gene family, this finding suggests a conserved genetic basis for the regulation of cell wall thickening and other functional properties of conducting cells across land plants.

## Conserved biochemical properties

An important aspect of vascular tissue development and its role in adaptation are the cell wall components that provide mechanical support under low turgor conditions or against the negative pressures associated with water conduction (de Vries and Archibald, 2018). Plant cell walls are composed mainly of cellulose and pectin polymers. These polymers are highly conserved throughout phylogeny but differ in their abundance and localization between embryophytes (Table 2; Popper and Tuohy, 2010; Niklas et al., 2017; Vanholme et al., 2019; Jamet et al., 2020). For example, mannan is relatively abundant in bryophytes and fern cell walls, yet almost absent from angiosperms (Plancot et al., 2019; Chernova et al., 2020), while xylan is more abundant in tracheophytes (Carafa et al., 2005). However, the most striking difference seems to be the absence of lignin polymers from all extant bryophytes (Popper and Tuohy, 2010).

**Table 2:** Cell wall components and polymers in land plant taxa. (A) Known cell wall components within the Charophyte (as common ancestors) which are found with other land plants and specific polymers present only in other land plants, "✓" indicated the presence of a specific polymer. (B) The presence of several known polymers and the abundance within different land plant taxa. Numbers correspond to references as follows: 1, Matsunaga et al. (2004); 2, Popper and Tuohy (2010); 3, Carafa et al. (2005); 4, Popper (2008); 5, Harris (2005).

A) Cell wall components in land plant taxa (1–5)					
Polymer	Charophytes	Bryophytes	Tracheophytes	Horsetails	Monocots
Cellulose	✓	✓	✓	✓	✓
Xylan	✓	✓	✓	✓	✓
Mannan	✓	✓	✓	✓	✓
Xyloglucan	✓	✓	✓	✓	✓
Arabinogalactan	✓	✓	✓	✓	✓
β-D-glucan				✓	✓
Lignin			✓	✓	✓
Silica		✓		✓	✓

B) Polymer presence and abundance in land plant taxa			
Polymer	Bryophytes	Ferns/Lycophytes	Seed plants
Rhamnogalacturonan-II (1,2)	Gametophyte (+)	Throughout plant (++)	Throughout plant (++)
Xylan (3)	Gametophyte (+)	Vascular and mechanical tissue (++)	Vascular and mechanical tissue (++)
Lignin (4)	/	Tracheids (++)	Tracheids (++)
Mannan (5)	Gametophyte (++)	Fibers (+++)	Seeds (+)

Lignin is a polymer of phenolic precursors derived from the phenylpropanoid pathway. In Arabidopsis, its synthesis is under control of VND6/7 NAC-domain proteins. Eleven core enzyme families are involved in the phenylpropanoid pathway, of which eight are dedicated to lignin synthesis [reviewed in Yao et al. (2021)]. Together, these enzymes create the three main lignin monomers: p-hydroxyphenyl (H), guaiacyl (G), and syringyl (S) monolignols. These monolignols can polymerize to form distinct classes of lignin (Weng and Chapple, 2010; Niklas et al., 2017). Throughout the tracheophyte lineage, there are distinct preferences for subclasses: H-lignin is abundant in seed-free vascular plants, G-lignin in gymnosperms, and S-lignin is only present in angiosperms and lycophytes (Weng et al., 2008).

In bryophytes, the major components of the phenylpropanoid pathway are conserved (Kriegshauser et al., 2021), including the enzyme families responsible for lignin monomer synthesis (Xu et al., 2009). This corresponds with the detection of H- and G-lignin monomers (Espiñeira et al., 2011) and the detection of lignin-specific epitopes in bryophytes (Ligrone et al., 2008). Only the enzyme necessary for S-lignin biosynthesis seems absent from bryophytes (Yao et al., 2021). However, the genomic conservation of these gene families does not directly imply conserved functions as some *P. patens* enzymes were shown to utilize a different substrate and, thus, potentially catalyze a different reaction, outside the lignin synthesis pathway (Renault et al., 2019).

Lignin is important in strengthening the cell wall by excluding water due to its aromatic nature. In bryophytes, the phenolic dimer lignan is present (Weng and Chapple, 2010). It is not unlikely that utilizing different compounds and/or a mixture of them can create lignin-like characteristics, especially as knowledge about the exact bryophyte cell wall composition and variety is still limited (Pfeifer et al., 2022). Additionally, it was shown that *Polytrichum commune* hydroids could withstand similar physical pressures and be as effective in water transport as tracheids (Brodribb et al., 2020). This indicates that the only shared functional characteristics of transporting tissues are related to their ability to transport by coping with the forces associated with this function and preventing embolisms, with or without lignin.

In an evolutionary context, lignin does not seem to be exclusively associated with fossilized plants that are classified as tracheophytes. The fossil record suggests a gradual evolution and incorporation of lignin into the transport system (Weng and Chapple, 2010). In *Aglaophyton*, a protracheophyte without true tracheids thought to bridge the bryophyte–tracheophyte gap (Cascales‐Miñana et al., 2019), lignin is only found outside the conductive tissues (Boyce et al., 2003). Similarly, the presence of thin-walled hydroids in bryophytes, unlike the tracheids with secondary wall thickenings of vascular plants, suggests that the functional constraints are dependent on the specific species and their needs. It could well be that bryophytes do not need a specific phenolic polymer like lignin in their conductive tissue, due to their growth habit.

## Discussion and outlook

The presence of a vascular system is generally considered a defining feature of tracheophytes, which grants them an adaptive advantage in terrestrial habitats by providing the combination of an efficient transport system and mechanical support. However, corresponding tissues in bryophytes show clear similarities in structure, function, organization, and molecular regulation. These similarities, considered together with differences between the transport systems in tracheophytes and bryophytes, point toward a gradual increase in complexity along the evolutionary history of transporting tissues and no clear distinction between the two groups. Important for this consideration is to acknowledge both differences and evolutionary graduality between tracheophyte clades: from lycophytes and monocots to angiosperms, in terms of their transporting tissues (Spicer and Groover, 2010; Spencer et al., 2021). Although the presence of lignin in tracheophytes would seem to represent a major discriminator, its functional role in tracheophytes is probably not unique when looking at bryophyte WCC (Brodribb et al., 2020) and should also be considered in the context of their ability to control water loss along with stomata and cuticle emergence (Merced and Renzaglia, 2017; Kong et al., 2020; Clark et al., 2022).

The notion that there is no strict distinction between the transport tissues of bryophytes and tracheophytes but, rather, evolutionary continuity, is in line with the overall picture of the evolution of plant tissues and organs that seem to redeploy existing regulatory machinery among lineages by gene co-option (see Plackett et al., 2015; Harrison and Morris, 2018; Motte et al., 2020), rather than incorporating entirely new pathways or networks (Bowles et al., 2020). However, the evolution of genetic pathways does not necessarily correspond with the evolution of morphological structures, as is evident from the independent evolutionary origins of roots, that express similar genes (Huang and Schiefelbein, 2015). Nonetheless, these extant and derived forms give no clear picture of the ancestral starting point. The fossil record has been (e.g. Edwards et al., 2022) and will continue to be a source of crucial information and surprising discoveries that will contribute to the picture of transporting tissue evolution. Current understanding of the fossil record suggests deep origin of transporting tissues (pre-dating the tracheophytes) and stepwise evolution. However, substantial gaps currently existing in the exploration of its deepest reaches withhold direct evidence of intermediate forms (missing links) between major living and extinct lineages that could provide meaningful answers to the key questions on the evolution of transporting tissues.

Whole-genome duplications are thought to play pivotal roles in evolution by providing opportunities for large scale neo-functionalization (Jiao et al., 2011; Soltis and Soltis, 2016). However, no apparent whole-genome duplication seems to have occurred between the bryophytes and early tracheophytes (Clark and Donoghue, 2018). This is consistent with a gradual evolution—as supported by the fossil record—based on successive co-option of genes, and could explain why most vascular regulators are present throughout the phylogeny of land plants (Bowles et al., 2022). The diversification of gene families in tracheophytes subsequent to their divergence from bryophytes may well have facilitated the diversification and elaboration of more extensive developmental programs, including those regulating secondary growth.

A more important distinction between all the discussed species and lineages relates to their ecological niches and distinct evolutionary trajectories. The presence of WCC and FCC among and within the bryophyte lineages does not exactly parallel patterns of phylogeny. This complicates the interpretation of both phylogenetic relationships among those lineages and evolutionary trajectories of transporting tissues. The absence of conducting cells or known regulators in some species may well reflect the distinct evolutionary paths those lineages followed from common ancestors and their ancestral character states. For instance, in *P. patens* some gene families show an increased rate of diversification while others have an increased rate of gene-loss (Rensing et al., 2008). If the ancestor of bryophytes was more tracheophyte-like, as hypothesized by Donoghue et al. (2021), possible gene losses for some processes in bryophytes (Harris et al., 2020) could explain the differences between bryophyte conductive tissue and tracheophyte vascular tissues.

Similarly, it is hard to generalize common traits of vascular tissues within the tracheophyte clade. For example, there is variability in morphology, molecular regulation, and cell wall compositions between lycophytes, ferns, and seed plants. However, particularly genetic and molecular studies have focused largely on a small number of angiosperm species, and knowledge in other clades is largely absent. As many genomes are now becoming available from highly relevant taxa among ferns (Marchant et al., 2019) and hornworts (Li et al., 2020), future genetic studies on these species will likely provide insights into transporting tissue function and evolution, currently not attainable by available data.

Based on the above considerations, one could consider the traditional dichotomy between bryophytes and tracheophytes as helpful from some perspectives, but also misleading from an evolutionary viewpoint. The usage of a different terminology for functionally similar and potentially homologous cell types and tissues between the two clades reinforces this dichotomy and complicates comparative discussions. Focusing on analogies and homology, the evolution of similar solutions to different problems, or even different solutions to similar problems, may help us better understand the contexts in which transporting tissues act, and the evolutionary forces acting upon them (see Outstanding questions). Using the terminology proposed here (see Figure 1) could act as a first step to minimize confusion and shift the focus of discussions on the many similarities between conductive tissues in bryophytes and vascular tissues in tracheophytes, instead of highlighting the perhaps more limited set of differences between them.

ADVANCES

- Combining molecular and fossil data reveals unexplored insights into the evolution of transporting tissues.
- Progress in molecular biology applications through technical advances in e.g. sequencing leads to the possibility of establishing new model species across the evolutionary tree of land plants.

OUTSTANDING QUESTIONS BOX

- How does transporting tissue develop and differentiate at the anatomical level, and how is it regulated at the molecular level in less characterized species from across the taxonomic breadth of land plants?
- With no living common ancestor, will it ever be possible to determine the evolutionary path and ancestral state of transporting tissues?
- Can new fossil discoveries or reanalysis of available fossils provide a better understanding of the changes in development associated with the evolution of transporting tissues?

## Funding

This work was funded by a grant from the Graduate School Experimental Plant Sciences (EPS) to S.W. and the Research Foundation—Flanders (FWO; Odysseus II G0D0515N) to B.D.R.

*Conflict of interest statement*. None declared.

## Notes

All the authors conceptualized and wrote the manuscript.

The author responsible for distribution of materials integral to the findings presented in this article in accordance with the policy described in the Instructions for Authors (https://academic.oup.com/plphys/pages/general-instructions) is: Dolf Weijers (dolf.weijers@wur.nl).
